# Informational Needs of Patients with Cancer: A Qualitative Content Analysis

**DOI:** 10.31557/APJCP.2019.20.2.557

**Published:** 2019

**Authors:** Zohreh Khoshnood, Mahlegha Dehghan, Sedigheh Iranmanesh, Masoud Rayyani

**Affiliations:** *Nursing Research Center, Razi Faculty of Nursing and Midwifery, Kerman University of Medical Sciences, Kerman, Iran. *

**Keywords:** Needs assessment, qualitative study, patients, nursing

## Abstract

**Section Title:**

One of the most important needs of patients with cancer is informational needs. Getting relevant information about the disease can lead to a proper decision making, better response to treatment, lower levels of anxiety, and higher levels of quality of life. Therefore, the current study aimed to determine the informational needs of patients with cancer in southeast Iran.

**Materials and Methods:**

This conventional qualitative content analysis was done using a descriptive-explorative design. Data collection was done through conducting deep semi-structured interviews from September 2017 to March 2018 in cancer treatment centers of Kerman University of Medical Science, Iran. Data saturation was achieved after interviewing with 13 patients during 15 interviews. The patients were asked to narrate their experience about informational needs of cancer patients. The following concepts were considered: the unit of analysis, meaning unit, condensation, code, sub-category, category, and main category.

**Results:**

The results of the present study showed that informational needs of these patients could be categorized under one main category called awareness-oriented needs, including three subcategories. These subcategories included lack of knowledge about the nature of the disease, inappropriate control of the disease due to lack of knowledge, and lack of knowledge about cancer treatment methods.

**Conclusion:**

Results showed that patients with cancer tended to know what is the cancer and the ways of cancer treatment and complications. Therefore, it is suggested to perform further studies cultivating the awareness of cancer patients in these areas. Therefore So, It should be noted that designing and implementation of this needs assessment provide a comprehensive way to consult and collaborate with health care professionals, patients, and their families.

## Introduction

The importance of cancer is obvious to everyone in light of the changes in its prevalence worldwide, and its impact on family, economy, and society (Biniaz et al., 2014). It is the second most common cause of mortality in the least-developed countries. The number of new cases was reported to increase by 70%, from 14 million in 2012 to 22 million over the next two decades (Drugs and Crime, 2010).

The diagnosis of cancer is emotionally devastating, and it takes a lot of energy from the patient and his whole family to adapt to new conditions (Kästel et al., 2011, Borji et al., 2018). Anxiety, depression, sense of guilt, anger, anxiety and other psychological pressures, hospitalizations, the high cost of treatment and social trauma are some of the problems which affect the person with cancer (McCarthy et al., 2009). In general, the examination of needs can include strategies that focus on identifying unresolved concerns of patients, and it is important to note that the needs of participants will be changed after being diagnosed with cancer (Biniaz et al., 2014, Borji et al., 2017). The studies carried out in various countries, including China (Lam et al., 2011), Australia (Boyes et al., 2012, Hodgkinson et al., 2007a, Hodgkinson et al., 2007b), Europe (Armes et al., 2009, Griesser et al., 2011, Schmid-Büchi et al., 2011), and Japan (Okuyama et al., 2011) have identified and categorized these needs. According to Boyes et al., (2012) these needs involves into five categories: psychological needs, health and information systems, daily physical activity, care and support, and sexual needs.

Lack of knowledge about the disease makes patient afraid of the disease recurrence, feel worried about death; moreover, it increases stress and affects negatively the patients’ and their families lives (Papadakos et al., 2017, Khoshnood et al., 2018c). The need for information is one of the most important standards of care and support in cancer patients (Husson et al., 2013). Getting relevant information about the disease can lead to a proper decision making in patients, better response to treatment, lower levels of anxiety and depression, higher levels of quality of life, and improved satisfaction with care and control (Davies et al., 2008). Recent studies have shown that informational needs of these patients are critical to take control of cancer (Mulcare et al., 2011). In addition, Husson et al., (2013) showed that the most important informational needs in cancer patients were the need to know about the disease, the diagnostic tests, and the cancer treatment. Other studies also showed that fulfilling the informational needs regarding the benefits of diagnostic and therapeutic tests led to better decision-making about initiating and continuing the treatment and also led to better compliance with the disease and reduction of psychological needs of these patients (Mulcare et al., 2011, Rutten et al., 2005). 

Given the needs expressed about caring for cancer patients, previous studied suggested that cancer, in addition to medical treatment, required comprehensive nursing care with compassion, competence, and conscience, which was introduced as the basic right of all patients and their families (sheet No, 2011; Yousefi et al., 2011). Nurses are the main caregivers or active attend of patients and face many challenges in caring for patients with a cancer diagnosis(Kendall, 2006, Khoshnood et al., 2018b). These people, as the main members of the health care team, spend a lot of time with patients at all stages of cancer treatment (Van Rooyen et al., 2008); therefore, nurses have the opportunity to pass their sense of being cared about cancer patients through their behaviors (Zamanzadeh et al., 2010). They also can have effective therapeutic communication with patients and they are able to conduct educational activities (Vafaee-Najar et al., 2012). According to Gilbert et al., effective communication between nurse and patient affects the patients’ satisfaction (Gilbert and Hayes, 2009). Therefore, training patient to be an integral part of nursing care is essential (Bagherian et al., 2017). This type of training is a dynamic and continuous process initiating from the admission and continuing up to patient’s discharge (Ghorbani et al., 2014).

According to the literature review, it is essential to prioritize nursing research on identifying the educational and informational needs of patients with cancer in Iran (Abdollahzadeh et al., 2014). Given that qualitative research can reveal the hidden dimensions of this problem, it is one way of studying phenomena within a culture. The latent content analysis seeks to focus on a person’s experience, previous understanding, and knowledge which are embedded in culture and religion. This study was conducted to determine the informational needs of patients with cancer in the southeast of Iran by using qualitative content analysis.

## Materials and Methods

This conventional qualitative content analysis was conducted using a descriptive-explorative design. Content analysis research aims to attain a condensed and broad description of a phenomenon (Graneheim and Lundman, 2004). Purposive sampling was used to choose among patients with cancer (leukemia, Non-Hodgkin’s Lymphoma, colon, pancreas, and breast cancer). Data collection was done from September 2017 to March 2018 in cancer treatment centers of Kerman University of Medical Science, Iran. The first participant was a patient with leukemia, and a key informant recommended one person who in turn, recommended another. A 3rd year Ph.D. candidate in nursing performed the interviews. Data saturation was achieved after interviewing with 13 participants during 15 interviews. Two participants were interviewed two times because some questions developed in line with the original question and the researchers needed to ask other questions by reading the interview to complete the previous information. To capture rich and diverse information, participants (patients, family members, and nurses) with different and rich experience about the research concept were invited for interviewing. Moreover, patients with different age, role, and work experience were chosen by the second researcher to achieve a wide range of information. In total, ten cancer patients, one family member of cancer patients (mother of one cancer patient), and two nurses who worked in the oncology department were interviewed.

Interviews were done in the preferred places and times declared by the participants (Cancer wards, their homes, or nursing department of Kerman University of Medical Science). The participants were asked to narrate their experiences of their informational needs related to their disease. Clarifying and encouraging questions were used such as: ‘Would you please explain more about your informational needs when your disease started?’, ‘what did you do when you felt some informational needs?’, and ‘Can you provide an example?’ The interviews were tape recorded, transcribed verbatim, and analyzed by the first author. The interviews lasted between 40 to 110 minutes.

This study was approved by the ethics committee of Kerman University of Medical Sciences (Ethical Code: ir.kmu.rec.1395.580). The aim of the study was explained to the participants, and they allowed the researchers to publish the findings by signing a written consent form. The study process was explained to the participants, and they were informed that they could withdraw from the study at any time. Confidentiality was guaranteed and no names or facts were stated in data. Speaking about their experiences of the disease had an emotionally charged nature and the researcher’s handled such risk by being attentive and sensitive to the interviewees’ emotional reactions.

The following concepts were considered important in performing conventional qualitative content analysis: a unit of analysis, meaning unit, condensation, code, sub-category, category, and main category (Graneheim and Lundman, 2004). The qualitative content analysis is based on the unit of analysis. According to Graneheim and Lundman (Graneheim and Lundman, 2004), unit of analysis is those interviews that are large enough to be considered as a whole and small enough to keep in mind as a context for the meaning unit during the analysis process. In our study, each interview was considered as a unit of analysis. After determining the unit of analysis, the text was divided into meaning units. Each meaning unit consisted of words, sentences, or paragraphs containing aspects related to each other through their content and context. In the next step, we condensed the meaning units, while still preserving the core. The condensed meaning units were then coded and sub-categories were created. The next step was to create categories that were the core features of qualitative content analysis. A category is a group of codes that are similar in a manifest level. A main category is a recurrent thread of underlying meaning running through codes and categories; it can be seen as an expression of the latent meaning of a text (Graneheim and Lundman, 2004). Although the analysis process was systematic, there was a back-and-forth movement between the whole and parts of the text.

**Table 1:** Main Category, Subcategories, Sub-Subcategories, and a Sample of Codes

main category	subcategories	Sub-Subcategories	A sample of codes
awareness-oriented needs	included lack of knowledge about the nature of the disease	- need to learn about the causes of the disease after hearing the diagnosis - Lack of awareness about how the disease progressed - ambiguities and blame on how to prevent the disease	I was diagnosed with non-Hodgkin's lymphoma. I really wanted to know more about it, but the resources were not enough and you could not find them easily. I went to the library but I didn’t get much information (P10)
	inappropriate control of the disease due to lack of knowledge	- Lack of awareness about the disease and the need for early referral to the physician - Lack of awareness in self-care during treatment - Lack of proper training and lack of awareness of the need for assistance from supporting organizations	They gave us a notebook, but it didn’t work. In both areas of food and resolving mental problems we needed to be trained, but there was no one who would give these trainings(P13)
	lack of knowledge about cancer treatment methods	- Need to know about existing treatments - Lack of knowledge about complementary and non-chemical methods of cancer treatment - Lack of knowledge about diagnostic methods and reason for doing them	I would love to know about the ways I can get better. Especially those who had the same disease as mine, it was really helpful. But I didn’t find anything(p14)

Four issues are generally used to describe various aspects of trustworthiness: credibility, confirmability, dependability, and transferability (Guba and Lincoln, 1989). Several techniques were used to enhance trustworthiness of current study. Such as Peer checking was done by the second researcher’s supervisors (the first and third researchers). The research team tried to collect data from different people with different experiences and different socio-demographic characteristics. Through frequent meetings between the second researcher and the supervisors, the study progress and the process were reported and discussed. Member checking was completed with some of the participants to achieve validation of the interpreted findings (codes and categories). Some of the faculty members checked the process of encoding and accessing categories (external checks). 

Some of limitations of this study were lack of the participants familiarity with the qualitative research methodology and the way of data collection was unusual for them and voice recording was not pleasant for some of the participants. The confidentiality of the information was assured to the participants. In particular, the researcher convinced them that he eliminated the voice recordings after the completion of the research. Furthermore, given that patients with cancer had a lot of psychological and physical problems and they felt tired because of fatigue and physical weakness, patients were preferably selected who had better disease statuses and had acute illness, or the duration of interviews were shortened and they were interviewed for two sessions.

## Results

13 persons participate in this study in 15 interviews. Three patients were male and seven were female. One of the participants was the patient’s family member and two of the participants were female nurses working in oncology wards. The age group of patients was 29-70 years old, the family member was 68 years old, and two nurses age was 34 and 38 years old. Among ten patients, two were single.

The results of the present study showed that informational needs of these patients could be classified under one main category called awareness-oriented needs. This category had three subcategories, including lack of knowledge about the nature of the disease, inappropriate control of disease due to lack of knowledge, and lack of knowledge about cancer treatment methods (Table 1).

*Lack of knowledge about the nature of the disease*

According to the experiences of the participants, the need to learn about the causes of the disease after being diagnosed, the lack of awareness about how the disease is progressed, and ambiguities of how to prevent it were the most important informational needs related to the nature of the disease.

Participants stated that as soon as they were diagnosed with cancer, they needed to obtain some information about the general nature of the illness. Most of them wanted to know what the disease is and how to prevent the disease. According to the statements and the experiences of the participants in this study, this awareness made the process of accepting the illness easier for them and prevented other consequent serious problems. According to findings, the desire to know the strategies for the management of the disease was one of the main concerns of patients. They believed that when they faced with the disease, they gradually became aware of its nature and blamed themselves that they could have prevented the deterioration of the disease if they had had more information. In the meantime, deficiencies in the training provided by the treatment team and the medical personnel were mostly expressed in interviews with patients. They found the training provided by health professional very weakly, saying that if these training courses had been provided to them more purposefully and comprehensively, many of the complications induced by the disease could be prevented.

“*I didn’t think at all that cancer is such a complicated disease, and that it can quickly kill the person (P4)*.”

“*I was diagnosed with non-Hodgkin’s lymphoma. I really wanted to know more about it, but the resources were not enough, and you could not access to them easily. I went to the library, but I could not get much information (P10) *»

*Inappropriate control of disease due to lack of knowledge*

In this sub-category lack of awareness of the disease and the need for early referral to the physician, the lack of awareness of self-care during treatment, the lack of proper training, and lack of awareness of the need for assistance from supporting organizations are the most important informational needs.

Patients, according to their own experience, believed that the training provided by caregivers, the mass media, and libraries was not sufficient. Furthermore, the existing educational resources provided in the hospitals, in the form of educational pamphlets, were not sufficient to meet the educational needs of these patients. Therefore, according to patients’ experiences, training should be provided by the treatment team or an informed person to help patients get information about the correct diets, how to deal with the disease, and how to strengthen the immune system as the mainstay of the educational needs of patients and their families in the area of disease management. Patients needed to know what the effects of the disease on the body and health of the patient are. They wanted to know what the effects of treatment, especially chemotherapy and radiation therapy are and what care they need during the treatment process. Therefore, providing the necessary strategies and training from the time of diagnosis and during the treatment process, especially during chemotherapy or radiotherapy is very helpful, and the defect in this information had led to poor management of symptoms in these patients.

“*They gave us a notebook, but it didn’t work. In both areas of food and resolving mental problems, we needed to be trained, but no one trained us (p13)*”

“*He can go walking, play sports, but we’re scared. We do not know anything about this platelet or something else. He wants to play sports, but we’re afraid that he’ gets worse (P12 is a sick mother)*”

“*For example, he must know that he must not eat anything for up to 2 hours, and then he must start the diet with apples, carrots or healthy fruits. For example, they can feed them with bean or something that is essential for blood formation. (P11 Oncology Nurse)* “

One of the other informational needs of patients was knowledge about supporting organizations. Some poor patients looked for a source of help, but because of the lack of information in this regard, they couldn’t get any help from these organizations. As one patient said:

“*It would be very good if there was a source that could help us. For example, the center of special disease is in Khorshid street, but it’s very limited, and we do not know to whom they help (P2)*»

*Lack of knowledge about cancer treatment methods*

Lack of knowledge about available treatments, lack of awareness of traditional, complementary and non-chemical methods of cancer treatment, and lack of knowledge about diagnostic techniques and the reason for each indication were the most important informational needs that these patients had experienced.

Patients expressed that information available in the media was not enough. When a person is diagnosed with cancer, he/she encounters plenty of uncertainties that create questions in his/her minds. According to our findings, patients faced with a lot of fear and anxiety, because they had very little information about cancer and its treatment; in other words, they experienced a mental crisis. During the illness and treatment, they were advised to learn about diagnostic tests and reasons for each indication, treatment methods and their effects on the body, and to consult with other patients suffering from the same disease and could dealt with the disease successfully.. Participants believed that such lack of awareness is due to inadequate information provided in the media and public gatherings.

“*I would like to know about the ways I can get better and talk to other patients with the same disease, it could be really helpful, but I didn’t find anyone (P14)*”

## Discussion

In the present study, informational needs of cancer patients were awareness of the nature of the disease, Its management, and different cancer treatments other than chemical treatments. According to previous studies, the most important informational needs of patients with head, neck, oral, pharyngeal, gynecologic, and gastrointestinal cancer, related to physical, medical, emotional, practical, social, and spiritual domains; respectively (Papadakos et al., 2017; Giuliani et al., 2016; Manne et al., 2016; Papadakos et al., 2012; Papadakos et al., 2015; Khoshnood et al., 2018a). On the other hand, some factors related to ways of providing information to patients. Husson et al., (2013) demonstrated that satisfaction with the received information was the most important factor associated with better illness perception. In addition, Mulcare et al., (2011) reported that patients with higher levels of anxious preoccupation were more likely to report a high need for action orientated information (dealing with potential problems).

According to our findings, the need to learn about the causes of their disease, the lack of awareness about how the disease is progressed, ambiguities of cancer prevention were the most important needs associated with the nature of the disease. In this regard, Mulcare et al., (2011) revealed that a patient who exhibited a high level of fighting spirit or anxious preoccupation would also have a high need for disease orientated information; whereas, a patient who exhibited a cognitively avoidant adjustment style would have low needs for disease orientated information. In addition, Papadakos et al., (2017) investigated the most highly rated informational needs in cancer patients. These needs were information about side effects prevention and management, the possible side effects induced by cancer treatment methods, and treatment of cancer.

The need to know about available treatments, lack of awareness of traditional and complementary medicine, non-chemical methods of cancer treatment, and knowledge about diagnostic methods and the reason for each indication were the main informational needs of patients in the present study. Patients at an advanced stage of the disease (III or IV) were more satisfied to receive more information about treatment and other services than patients at earlier stages of the disease (I and II) (Husson et al., 2013).

In contrast with the findings of the present study, spiritual domain received the lowest score concerning the informational needs in a previous study (Papadakos et al., 2015). These differences regarding information needs can be due to difference in socio-demographic and clinical characteristics. Given that Iran a religious country (Dehghan Nayeri, 2015) and due to Iranian religious orientation and traditional lifestyle, they tend to use more complementary therapies.

Based on the results of the present study, patients with cancer needed to have some information about the nature of cancer as well as its management and treatment. Given the advancement of technology and increase of community information especially among those who suffer from a disease, the needs of patients also have changed, searching for ways to treat their disease. Accordingly, the informational needs of patients have altered. Therefore, training about cancer, its therapeutic methods, , modern treatment methods, and counseling can be an effective step in reducing the informational needs of these patients and their families. Therefore, further studies are suggested in this regard. Also, they want to know how cancer is treated by traditional medicine and the development of protocols in this field. So, It should be noted that designing and implementation of this needs assessment provide a comprehensive way to consult and collaborate with health care professionals, patients, and their families.

## Conflict of interests and funding

There is no conflict of interest to declare. The authors received no financial support for the research and publication of this article and this article has not been published in another journal.
